# Amyloid precursor protein (APP) and amyloid β (Aβ) interact with cell adhesion molecules: Implications in Alzheimer’s disease and normal physiology

**DOI:** 10.3389/fcell.2022.969547

**Published:** 2022-07-26

**Authors:** Grant Pfundstein, Alexander G. Nikonenko, Vladimir Sytnyk

**Affiliations:** ^1^ School of Biotechnology and Biomolecular Sciences, University of New South Wales, Sydney, NSW, Australia; ^2^ Department of Cytology, Bogomoletz Institute of Physiology, Kyiv, Ukraine

**Keywords:** cell adhesion molecule (CAM), Alzheimer’s disease, amyloid precursor protein (APP), amyloid-beta, immunoglobulin superfamily, integrin, prion protein (PrP), neurexin

## Abstract

Alzheimer’s disease (AD) is an incurable neurodegenerative disorder in which dysfunction and loss of synapses and neurons lead to cognitive impairment and death. Accumulation and aggregation of neurotoxic amyloid-β (Aβ) peptides generated *via* amyloidogenic processing of amyloid precursor protein (APP) is considered to play a central role in the disease etiology. APP interacts with cell adhesion molecules, which influence the normal physiological functions of APP, its amyloidogenic and non-amyloidogenic processing, and formation of Aβ aggregates. These cell surface glycoproteins also mediate attachment of Aβ to the neuronal cell surface and induce intracellular signaling contributing to Aβ toxicity. In this review, we discuss the current knowledge surrounding the interactions of cell adhesion molecules with APP and Aβ and analyze the evidence of the critical role these proteins play in regulating the processing and physiological function of APP as well as Aβ toxicity. This is a necessary piece of the complex AD puzzle, which we should understand in order to develop safe and effective therapeutic interventions for AD.

## Introduction

### Alzheimer’s disease and the amyloid hypothesis

Alzheimer’s disease (AD) is an incurable neurodegenerative disorder in which progressive synapse loss and neuronal dysfunction are followed by neuronal death in the brain. People with AD suffer from severe cognitive impairments and eventually die as a result of the disease. AD is the seventh leading cause of death in the United States (Alzheimer’s Association, 2022). Its global economic burden, together with other dementias, is estimated to be nearly $1 trillion (USD) per annum (Prince et al., 2015). Despite decades of research, effective therapeutic interventions for AD remain elusive, presenting a considerable problem given the increasingly aging population of our world today.

The amyloid hypothesis has been central to our understanding of AD for the past three decades. It proposes that aggregates of the amyloid-β (Aβ) peptide, the primary component of senile plaques formed in brains of people with AD, are the main causative agents in AD pathogenesis (Hardy and Allsop, 1991; Selkoe, 1991; Hardy and Higgins, 1992; Selkoe and Hardy, 2016; Karran and De Strooper, 2022). These Aβ aggregates bind to cell surface receptors on neurons and glial cells, inducing synaptic dysfunction and neuroinflammation, triggering aberrant intracellular signaling cascades that lead to hyperphosphorylation of the microtubule associated protein tau, causing disruption of axonal transport, oxidative damage, breakdown of homeostasis, and resulting in neurotoxicity (LaFerla et al., 1995; Sakono and Zako, 2010; Xia et al., 2016; Yin et al., 2017; Sushma and Mondal, 2019). The gradual loss of synapses and neurons is responsible for cognitive impairment, memory loss, and, eventually, death.

### Amyloid precursor protein (APP) and its processing

**FIGURE 1:**
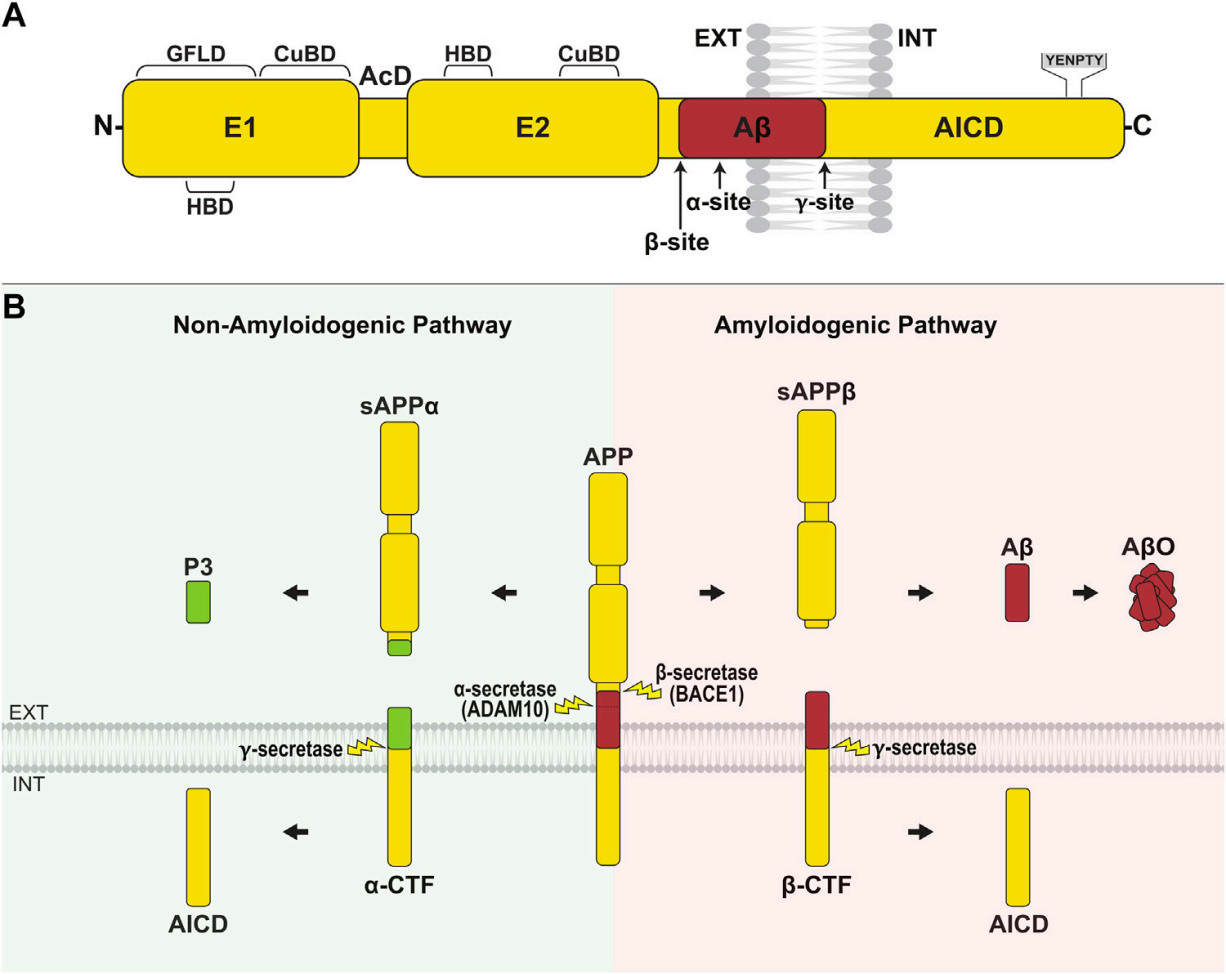
APP structure and processing. **(A)** APP is composed of a large N-terminal extracellular domain, transmembrane region, and short cytoplasmic tail. The extracellular domain comprises two rigidly folded regions, E1 and E2, joined by an acidic domain (AcD). E1 contains a heparin-binding domain (HBD) within a larger growth factor-like domain (GFLD), and a copper/zinc-binding domain (CuBD). E2 comprises the second HBD and CuBD. The juxtamembrane region contains the α- and β-cleavage sites, while the γ-cleavage site is located within the transmembrane domain. The C-terminal intracellular domain (AICD) contains the YENPTY sequence, which binds cytosolic adaptor proteins. **(B)** APP is primarily processed along two opposing pathways. In amyloidogenic processing, APP is cleaved by β-secretase (BACE1) at the N-terminus of Aβ, generating sAPPβ and the membrane-bound β-CTF. Subsequent γ-secretase cleavage of β-CTF releases the Aβ peptide into the extracellular/lumenal space and AICD into the cytosol. Aβ peptides aggregate and form oligomers (AβO). In non-amyloidogenic processing, APP is cleaved by α-secretase (ADAM10) within the Aβ region, producing sAPPα and α-CTF. Ensuing cleavage of α-CTF by γ-secretase liberates P3 into the extracellular/lumenal space and AICD into the cytosol.

Aβ peptides are generated *via* the proteolysis of amyloid precursor protein (APP). APP is a type I transmembrane glycoprotein with a large N-terminal extracellular domain, transmembrane region, and short intracellular tail (Figure 1A). APP is encoded by a single gene on chromosome 21, and alternative splicing generates three main isoforms, APP695, APP751, and APP770, denoted by their amino acid length. The extracellular part of all isoforms is comprised of two rigidly folded domains, E1 and E2, joined by a flexible acidic domain (AcD) and connected to the transmembrane region by a mostly unstructured juxtamembrane linker (Coburger et al., 2013; Coburger et al., 2014) (Figure 1A). E1 contains a heparin-binding domain (HBD) within a larger growth factor-like domain (GFLD), as well as a copper/zinc-binding domain (CuBD). E2 contains the second HBD and CuBD (Dahms et al., 2012). The extracellular domain mediates homophilic trans-interactions, where an APP molecule on one cell binds to an APP molecule on an adjacent cell, allowing APP to function as a cell adhesion molecule (Soba et al., 2005; Stahl et al., 2014). The extracellular domain also binds other ligands such as epidermal growth factor (EGF) (da Rocha et al., 2021) and reelin (Hoe et al., 2009), and acts as a ligand itself after being shed into the extracellular space where it interacts with other receptors (Caillé et al., 2004). The single-pass transmembrane domain is involved in cholesterol binding (Barrett et al., 2012), while the short intracellular tail contains a YENPTY motif that binds cytoplasmic adaptor proteins such as Dab1 and Mint, which mediate APP-dependent signaling (Borg et al., 1996; King et al., 2003; Schettini et al., 2010). The Kunitz protease inhibitor (KPI) domain is present in the two longer isoforms, APP751 and APP770, and the OX-2 sequence is contained in APP770 (Tanzi et al., 1987; Sandbrink et al., 1996). APP is widely expressed in many tissues. APP695 is the major neuronal isoform, while APP751 and APP770 are highly expressed in non-neuronal cells (Rohan de Silva et al., 1997).

APP undergoes complex proteolytic processing, yielding a number of biologically active fragments. Processing along the amyloidogenic pathway is initiated by β-site APP cleaving enzyme 1 (BACE1), which cleaves APP at the amino terminus of the Aβ region, producing sAPPβ and the membrane-bound β-C-terminal fragment, β-CTF (Vassar et al., 1999; Yan et al., 1999; Zhang et al., 2011) (Figure 1B). In the non-amyloidogenic pathway, α-secretase (ADAM10; a disintegrin and metalloproteinase 10) cleaves APP between Lys 16 and Leu 17 of the Aβ region, preventing the formation of Aβ and releasing sAPPα and α-CTF instead (Lammich et al., 1999; Asai et al., 2003; Kuhn et al., 2010). The cell surface or trans-Golgi network accumulation of APP favors non-amyloidogenic processing (Parvathy et al., 1999; Tan and Gleeson, 2019), whereas the retention of APP in endocytic compartments promotes amyloidogenic processing (Das et al., 2016). In both pathways, γ-secretase, a complex composed of presenilin 1 (PS1), nicastrin, anterior pharynx-defective 1 (APH-1), and presenilin enhancer 2 (PEN-2), cleaves the membrane-tethered CTFs yielding Aβ from β-CTF and P3 from α-CTF, as well as the APP intracellular domain (AICD) (Figure 1B). In the amyloidogenic pathway, γ-secretase produces peptides of varying length with Aβ_40_, denoted by amino acid length, being most abundant and Aβ_42_ being most aggregation prone (Jarrett et al., 1993; Hamley, 2012; Chen et al., 2017). A shift towards the generation of Aβ_42_ over Aβ_40_, or an imbalance in the overall production and clearance of Aβ peptides predisposes to the formation of neurotoxic oligomers (AβO), fibrils (AβF) and plaques (Masters et al., 1985; Bharadwaj et al., 2009; Mawuenyega et al., 2010). In addition, there are several non-canonical pathways through which APP can be processed, including proteolysis by η-secretase, δ-secretase, meprin, and caspases (Andrew et al., 2016).

APP is a member of a small family of proteins consisting of APP and APP-like proteins 1 and 2 (APLP1 and APLP2) (Heber et al., 2000; Walsh et al., 2007). Although structurally very similar to APP, APLPs lack the Aβ region and are thus non-amyloidogenic. As a whole, members of the APP family share poorly understood roles in synaptic plasticity, synaptogenesis, neurite outgrowth, learning, and memory (Small et al., 1994; Müller and Zheng, 2012; Klevanski et al., 2015).

Many therapeutic interventions have targeted Aβ and the proteases responsible for its generation, however thus far, all attempts have failed to demonstrate reasonable efficacy and are associated with worsening cognition and other side effects (Kumar et al., 2018; Hampel et al., 2021; Karran and De Strooper, 2022). These failures can be partly attributed to an incomplete understanding of the complex cell biology underlying: *1*) the processing of APP, *2*) the normal physiological function of APP and its proteases, and *3*) the mechanisms of Aβ-induced toxicity. A class of proteins intimately involved in these poorly understood processes are cell adhesion molecules (CAMs), a vast category of cell surface proteins that mediate adhesion of cells to one another and to the extracellular matrix (ECM). These broadly include CAMs of the immunoglobulin superfamily (IgSF), integrins, cadherins, selectins, and other uncategorized proteins that possess adhesive function, including APP itself. CAMs are of particular interest in amyloid-dependent AD pathology as their structure and cell surface localization make them well-suited for interactions with both APP and Aβ, many of which have been found to date. Multiple gene ontological analyses have also described cell adhesion as an affected pathway in AD (Blalock et al., 2004; Cui et al., 2018; Wang and Li, 2021; Shu et al., 2022).

Thus, in this review, we summarize the current knowledge surrounding the interactions of CAMs with APP and Aβ and how CAMs influence the processing and physiological function of APP as well as their role in Aβ toxicity. This breadth of knowledge is a necessary piece of the complex AD puzzle, which we should better understand to enable the development of safe and effective therapeutics for the treatment and prevention of AD.

## Interactions of CAMs with APP and the role that CAMs play in the amyloidogenic processing of APP and Aβ toxicity

### APP family

As APP family proteins are themselves CAMs, we first discuss the role that homo- and hetero-dimerization of the members of this family plays in APP processing, functioning, and Aβ toxicity.

#### APP homodimerization influences its processing

**FIGURE 2:**
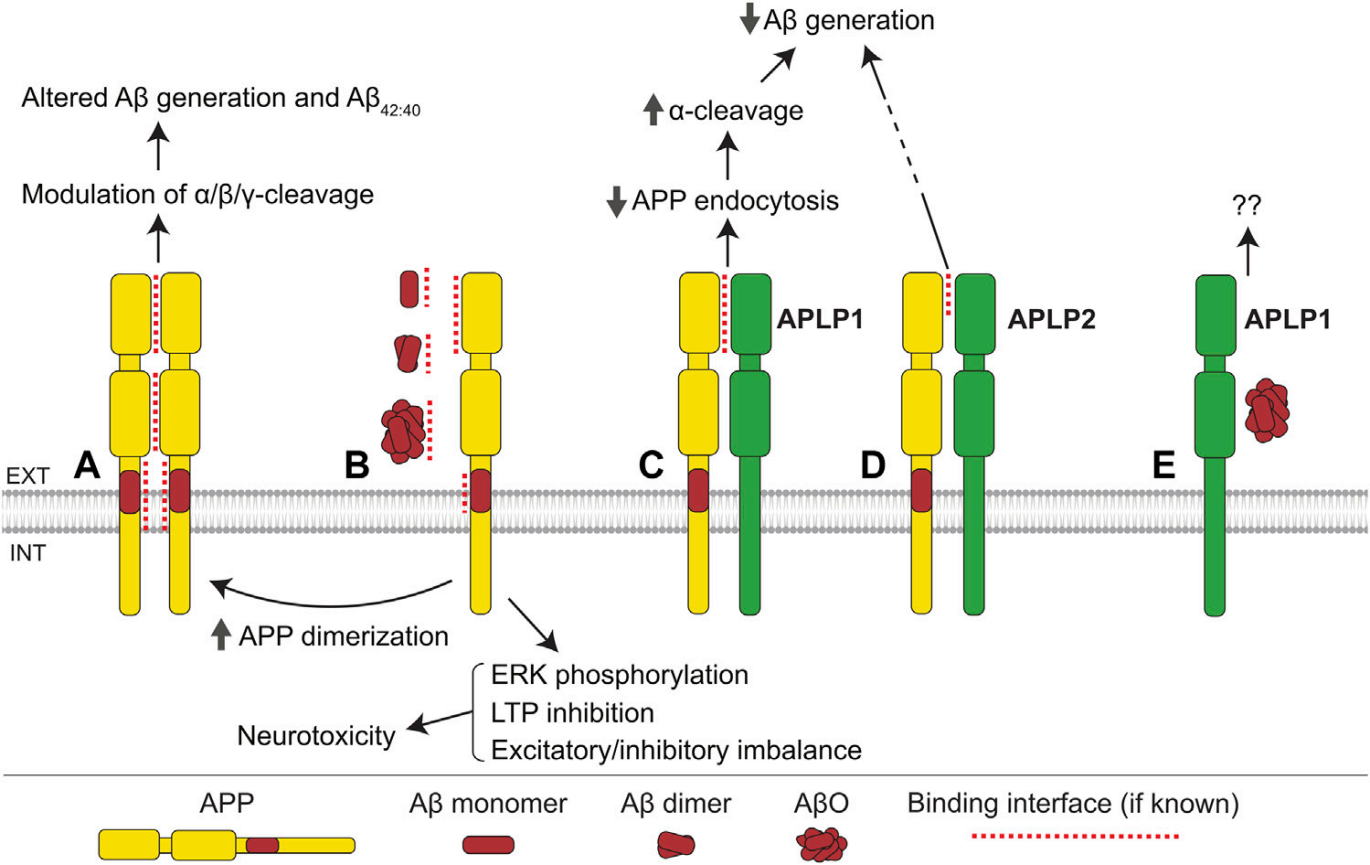
APP family interactions. **(A)** Interactions between E1, E2, and transmembrane domains of APP mediate formation of homodimers. Homodimerization influences the α-, β-, and γ-cleavage of APP, Aβ generation, and the ratio of Aβ_42/40_. **(B)** Monomers, dimers, and oligomers of Aβ bind to the E1 domain and cognate Aβ region of APP. The interaction with Aβ monomers and dimers promotes APP homodimerization, while the APP-AβO interaction induces ERK phosphorylation, inhibits long-term potentiation (LTP), and leads to excitatory/inhibitory imbalance, ultimately resulting in neurotoxicity. **(C)** APLP1 interacts with the E1 domain of APP, suppressing APP endocytosis, increasing cell surface levels of APP, and thereby facilitating the α-cleavage of APP, and consequently reducing Aβ generation. **(D)** APLP2 interacts with APP and reduces Aβ generation *via* an unknown mechanism. **(E)** APLP1 interacts with AβOs with unknown consequence.

APP interacts with itself forming homodimers in the mouse brain (Schmidt et al., 2012; Herr et al., 2017) and numerous cell lines, with at least four regions of this molecule, namely the GFLD of E1 (Kaden et al., 2009), CuBD of E1 (Noda et al., 2013), E2 (Wang and Ha, 2004), and TMD (Munter et al., 2007; Sato et al., 2009), being engaged in dimer formation (Figure 2A and Table 1). The impact of dimerization on APP processing is complex and remains controversial. Several reports suggest that APP dimerization modulates the balance between its α- and β-cleavage. For example, inhibition of APP dimerization using an APP-E1-derived peptide leads to an increase in sAPPα levels and reduction in sAPPβ and Aβ levels in SH-SY5Y cells (Kaden et al., 2008). Similarly, small molecule inhibitors of APP dimerization reduce sAPPβ and Aβ levels in HEK293 cells without affecting Aβ_42/40_ ratio (So et al., 2012), as well as favoring sAPPα over sAPPβ production in CHO and B103 cell lines (Libeu et al., 2012). Together, these findings suggest that APP dimerization promotes BACE1-mediated cleavage of APP. However, other studies report that APP dimerization in CHO cells leads to an increase in sAPPα levels and decrease in sAPPβ levels (Decock et al., 2015), while statin-induced TMD dimerization in iPSC-derived neurons reduces both sAPPα and sAPPβ levels, and decreases production of Aβ (Langness et al., 2021). In addition, a number of studies suggest that APP dimerization influences its γ-secretase-mediated cleavage. For example, mutationally induced dimerization of the juxtamembrane region of APP leads to an increase in Aβ levels without affecting sAPPα or sAPPβ levels, suggesting that the γ-secretase cleavage efficiency of APP-CTFs may be influenced by APP dimerization (Scheuermann et al., 2001). The preferred γ-cleavage site within APP may also be affected by the mode of dimerization, as copper-induced APP dimerization *via* the CuBD of E1 favors production of Aβ_40_ over Aβ_42_ while TMD dimerization promotes generation of Aβ_42_ over Aβ_40_ (Munter et al., 2007; Noda et al., 2013). It has also been suggested that juxtamembrane domain/TMD dimerization may predispose Aβ to form dimers and oligomers, which may in turn affect the levels and toxicity of Aβ (Scheuermann et al., 2001). A possible explanation for these controversies may lie in the fact that various regions of APP contribute to dimerization, and it is quite probable that variations in the contact sites involved may influence APP processing in different ways.

**TABLE 1:** APP-interacting cell adhesion molecules and their role in APP processing and physiological function.

APP-interacting protein		Function of interaction	Cell type/tissue(s) displaying interaction	Reference(s)
APP family	APP	Alters Aβ generation and Aβ_42/40_ ratio; May modulate α-, β-, and γ-cleavage	Mouse brain, HEK293, CHO, SH-SY5Y, B103, iPSC-derived neurons	Munter et al. (2007); Kaden et al. (2008); Libeu et al. (2012); Schmidt et al. (2012); So et al. (2012); Noda et al. (2013); Decock et al. (2015); Herr et al. (2017); Langness et al. (2021)
	APLP1	↓ APP endocytosis; ↑ α -cleavage; ↓ β-cleavage; ↓ Aβ generation; ↓ Aβ_42:40_; ↓ APLP1 surface levels	Mouse brain, HEK293, SH-SY5Y, COS7	Soba et al. (2005); Neumann et al. (2006); Bai et al. (2008); Kaden et al. (2009)
	APLP2	↓ Aβ generation; ↓ Aβ_42:40_	Mouse brain,; HEK293	Soba et al. (2005); Bai et al. (2008); Kaden et al. (2009)
IgCAMs	NCAM1	↓ Aβ generation; ↑ ERK phosphorylation; ↑ Neurite outgrowth	Mouse brain, CHO, COS7	Chen and Dou, (2012); Chen et al. (2016)
	Fasciclin 2	↑ Synapse formation	*D. melanogaster* body-wall muscle	Ashley et al. (2005)
	NgCAM	↑ APP and αCTF levels; ↑ Axon growth	Chick brain, chick retinal ganglion cells, HEK293T	Osterfield et al. (2008)
	Neurofascin	Unknown	Mouse brain	Bai et al. (2008)
	Contactin-1	↑ α -cleavage; ↓ β-cleavage; ↓ Aβ_42_ levels; ↓ Aβ_42:40_	Mouse brain	Bai et al. (2008); Puzzo et al. (2015)
	Contactin-2	May modulate α/β cleavage; ↑ γ-cleavage; ↓ Neurogenesis; ↓ TGFβ2-induced cell death	Mouse brain, CHO, F11	Ma et al. (2008); Tachi et al. (2010)
	Contactin-3	Unknown	Chick brain and *in vitro*	Osterfield et al. (2008); Peng et al. (2019); Karuppan et al. (2022)
	Contactin-4	↑ APP and αCTF levels; ↑ Axon-target matching	Mouse brain, chick brain, mouse retinal ganglion cells, HEK293	Osterfield et al. (2008); Osterhout et al. (2015); Peng et al. (2019); Karuppan et al. (2022)
	Contactin-5	Unknown	*In vitro*	Peng et al. (2019); Karuppan et al. (2022)
Integrins	α3	Unknown	Mouse brain	Hoe et al. (2009)
	β1	↑ APP surface levels; ↑ Monocyte activation; May increase α-cleavage; Alters cell adhesion; Alters neurite outgrowth	Mouse brain, HEK293, CHO, U937, human umbilical vein endothelial cells, THP-1 monocytes	Ghiso et al. (1992); Sondag and Combs, (2004); Young-Pearse et al. (2008); Hoe et al. (2009); Rice et al. (2013); Ristori et al. (2020)
Cadherins	N-cadherin	↑ APP homodimerization; ↑ sAPPβ and Aβ production	Mouse brain, HEK293	Asada-Utsugi et al. (2011)
	E-cad/CTF2	↑ APP-CTF lysosomal degradation; ↓ Aβ production	CHO	Agiostratidou et al. (2006)
	Calsyntenin-1	Stabilizes APP-Mint2 interaction; ↓ β-cleavage; ↓ Aβ production	Mouse brain, HEK293	Araki et al. (2003); Bai et al. (2008); Takei et al. (2015); Gotoh et al. (2020)
	Calsyntenin-3	Unknown	Mouse brain	Bai et al. (2008)
Neurexins	1α, 2	Unknown	Mouse brain	Norstrom et al. (2010)
	Caspr-1	May alter APP stability/processing; May alter Aβ production	Mouse brain, HEK293, CHO	Hur et al. (2012); Fan et al. (2013)
Prion Protein	PrP^c^	↑ Cell adhesion	Human brain, mouse brain, zebrafish, N2a	Schmitt-Ulms et al. (2004); Bai et al. (2008); Kaiser et al. (2012); Ulbrich et al. (2018)
LRR-CAMs	LRRTM3	↑ β-cleavage; ↑ Aβ production	SH-SY5Y, HEK293T	Majercak et al. (2006); Lincoln et al. (2013)
	FLRT1,3	Unknown	HEK293T	Yu et al. (2016)

#### Interactions of APP with other APP family proteins influence APP processing

APP interacts with APLP1 in the mouse brain (Soba et al., 2005; Bai et al., 2008). This interaction is mediated partially by the E1 domains of APP and APLP1 (Figure 2C), but other regions are suggested to be also involved (Kaden et al., 2009). While APP is mostly found intracellularly, APLP1, in contrast, is predominantly localized at the cell surface and has a much slower rate of endocytosis compared to APP (Kaden et al., 2009; Schilling et al., 2017). Accordingly, APP-APLP1 interactions are reported to suppress endocytosis of APP, increasing its α-cleavage and reducing its β-cleavage in HEK293 cells (Neumann et al., 2006). In agreement, the co-expression of APP and APLP1 in SH-SY5Y cells results in reduced Aβ generation compared to cells transfected with APP only (Kaden et al., 2009). APP also influences the subcellular distribution of APLP1, reducing its levels at the cell surface (Kaden et al., 2009). Given the emerging role of APLP1 as the primary cell adhesion molecule of the APP family (Mayer et al., 2016; Dunsing et al., 2017; Schilling et al., 2017; Onodera et al., 2021), these effects of APP on APLP1 distribution provide evidence supporting the involvement of APP in cell adhesion regulation. In the mouse brain, APP also binds to APLP2 *via* its GFLD of E1 (Soba et al., 2005; Kaden et al., 2009). Similarly to APLP1, APLP2 reduces Aβ generation in HEK293 cells when it is co-expressed with APP (Kaden et al., 2009) (Figure 2D). APLPs preferentially reduce Aβ_42_, rather than Aβ_40_ levels, suggesting that APP/APLP interactions affect the γ-secretase-mediated cleavage of APP-CTFs (Kaden et al., 2009).

#### APP interacts with Aβ and mediates Aβ toxicity

APP interacts with Aβ monomers, oligomers, and fibrils, acting as a receptor for Aβ, which mediates Aβ-induced toxicity (Figure 2B and Table 2). Soluble Aβ was found to bind to the Aβ region of APP at the cell surface thereby inducing cell death in N2a cells (Shaked et al., 2006). This effect was dependent on the YENPTY motif in the APP intracellular domain, which interacts with a number of cytoplasmic proteins involved in intracellular signaling, suggesting that Aβ induces intracellular signaling pathways *via* APP. Consistent with this idea, AβO treatment of B103 cells increases Ras levels and ERK phosphorylation, both of which are dependent on APP expression (Kirouac et al., 2017). ERK induces hyperphosphorylation of tau and thereby can mediate the APP-dependent AβO toxicity (Guise et al., 2001; Siano et al., 2019). In APP−/− mice, AβO binding to synapses is reduced and AβO effects on long-term potentiation (LTP) and the balance of excitatory/inhibitory activity are attenuated (Wang et al., 2017). Cultured APP−/− neurons are less vulnerable to Aβ-induced toxicity compared to wild-type neurons (Lorenzo et al., 2000). Aβ monomers and dimers also bind to the E1 domain of APP, increasing APP homodimerization and influencing neurotransmitter release probability (Fogel et al., 2014). In addition, AβFs bind to APP leading to an increase in APP levels at the cell surface, thereby facilitating the binding of Aβ to APP (Lorenzo et al., 2000; Heredia et al., 2004). APP-Aβ interactions promote homodimerization of APP, which may in turn stimulate amyloidogenic processing of APP to a greater extent (Fogel et al., 2014). APLP1 has also been identified as a binding partner of AβO, however, the functional significance of this interaction remains to be determined (Figure 2E).

**TABLE 2:** Aβ-interacting cell adhesion molecules and their role in Aβ toxicity.

Aβ-interacting protein		Aβ species	Function of interaction	Cell type/tissue(s) displaying interaction	Reference(s)
APP family	APP	Monomers; Oligomers; Fibrils	↑ Neurotoxicity	Mouse brain, rat cortical neurons, N2a, B103	Lorenzo et al. (2000); Heredia et al. (2004); Shaked et al. (2006); Fogel et al. (2014); Kirouac et al. (2017); Wang et al. (2017)
			↑ Ras levels		
			↑ ERK phosphorylation		
			↓ LTP		
			↑ APP dimerization		
	APLP1	Oligomers	Unknown	COS7	Laurén et al. (2009)
IgCAMs	NCAM2	Oligomers	↑ NCAM2 shedding; ↑ Synapse loss	Mouse brain	Leshchyns’ka et al. (2015)
	L1	Monomers; Low-MW oligomers	↓ High-MW AβOs	Mouse brain and *in vitro*	Djogo et al. (2013)
			↓ Plaque load		
			↓ Synapse loss		
			↓ Astrogliosis		
Integrins	α1, α2, αV, β1	Monomers; Oligomers; Fibrils	↑ Neurotoxicity	Human fetal cortical cultures, mouse brain, rat oligodendrocytes, THP-1 monocytes, rat mast cells, HT22, BV-2, SH-SY5Y, IMR-32	Sabo et al. (1995); Matter et al. (1998); Bamberger et al. (2003); Anderson and Ferreira, (2004); Bozzo et al. (2004); Koenigsknecht and Landreth, (2004); Wright et al. (2007); Niederhoffer et al. (2009); Han et al. (2013); Woo et al. (2015); Quintela-López et al. (2019); Ortiz-Sanz et al. (2020)
			↑ Cofilin activation		
			↑ Mitochondrial dysfunction		
			↑ ROS generation		
			↑ Apoptosis		
			↑ MAPK activation		
			↑ Dendritic complexity and spine density		
			↑ Oligodendrocyte survival		
			↑ Microglial activation		
			↑ Aβ phagocytosis		
			↓ Cell surface integrins		
			↓ Cell adhesion		
Neurexins	1, 2, 3	Oligomers	↓ Presynaptic differentiation	Human brain (*ex vivo*), rat hippocampal neurons, COS-7, HEK293	Brito-Moreira et al. (2017); Naito et al. (2017)
			↓ Neurexin-1β axonal cell surface levels		
			↑ Oxidative stress		
			↑ Synapse loss		
			↑ Memory impairment		
		Fibrils	Unknown	CSF of AD patients	Rahman et al. (2018)
Neuroligins	1	Oligomers	↑ Oxidative stress	Human brain (*ex vivo*), mouse brain	Dinamarca et al. (2015); Brito-Moreira et al. (2017); Dufort-Gervais et al. (2020)
			↑ Synapse loss		
			↑ Memory impairment		
	2	Oligomers	Unknown	Rat hippocampal neurons	Dinamarca et al. (2015); Brito-Moreira et al. (2017)
Prion protein	PrP^c^	Oligomers	↓ LTP	Human brain, mouse brain, COS-7, SH-SY5Y, HEK293	Laurén et al. (2009); Barry et al. (2011); Caetano et al. (2011); Larson et al. (2012); Rushworth et al. (2013); Peters et al. (2015); Gunther et al. (2019)
			↑ Intracellular Ca^2+^		
			↑ Fyn activation		
			↑ Tau hyperphosphorylation		
			↑ Synaptotoxicity		
			↑ Memory impairment		
			↑ Cell surface PrP^c^		
			↓ PrP^c^-mediated BACE1 inhibition		

### Immunoglobulin superfamily of CAMs

Members of the immunoglobulin superfamily (IgSF) of CAMs are plasma membrane-attached glycoproteins characterized by the presence of immunoglobulin-like (Ig) repeats within their extracellular domains. IgSF CAMs mediate calcium-independent homophilic adhesion between cells where identical molecules on membranes of adjacent cells bind to each other. IgSF CAMs also heterophilically interact in *cis* with a number of other cell surface receptors located within the same membrane or bind in *trans* to the cell surface receptors on membranes of other cells. These proteins play important roles in regulating neuronal development and synaptic functions (Maness and Schachner, 2007; Sytnyk et al., 2017) and have been implicated in AD (Leshchyns'ka and Sytnyk, 2016). The role that APP and Aβ play in regulating the functions of these proteins and effects of these proteins on the amyloidogenic processing of APP and Aβ toxicity are reviewed below.

#### Neural cell adhesion molecules (NCAMs)

**FIGURE 3:**
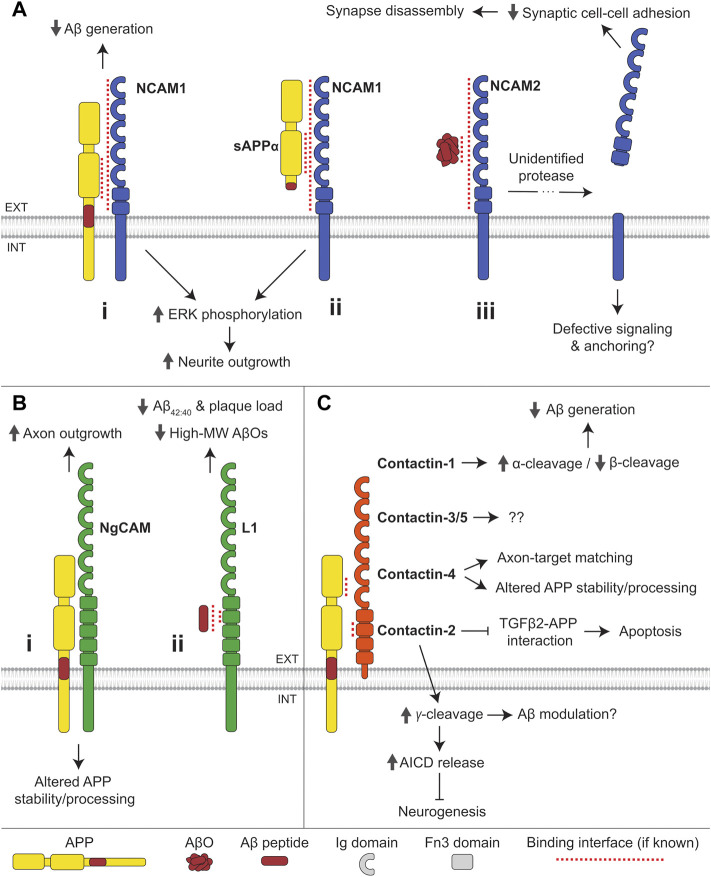
Interactions of IgSF CAMs with APP and Aβ. **(A) (i)** NCAM1 interacts with the E2 domain of APP and reduces Aβ generation. Binding of APP or **(ii)** sAPPα to NCAM1 induces ERK phosphorylation and promotes neurite outgrowth. **(iii)** AβOs bind to NCAM2 triggering shedding of its extracellular domain by an unidentified protease, causing synapse disassembly. **(B) (i)** The chicken ortholog of L1, NgCAM, interacts with APP and regulates APP stability and processing. Binding of APP to NgCAM promotes axon outgrowth in retinal ganglion cells. **(ii)** Aβ peptides bind to the second Fn3 domain of L1. L1 reduces Aβ_42:40_ ratio and the formation of high molecular-weight (MW) AβOs in favor of monomers, dimers, and tetramers. **(C)** APP interacts with all contactin family members except for contactin-6. The E1 CuBD of APP binds to the second Fn3 domain of contactin. Contactin-1 promotes α-cleavage and reduces β-cleavage of APP, thereby reducing Aβ generation. Contactin-2 promotes γ-cleavage of APP, thereby increasing AICD release and inhibiting neurogenesis. Contactin-2 also inhibits the binding of APP to TGFβ2 and reduces apoptosis induced by APP-TGFβ2 interactions. Contactin-4 regulates APP stability and processing and is required for APP-dependent axon-target matching. The function of APP interactions with contactin-3 and -5 remains undetermined.

NCAMs belong to a sub-family of CAMs within the IgSF. The group consists of NCAM1 (originally designated NCAM) and the lesser known NCAM2 (also designated OCAM) (Winther et al., 2012; Weledji and Assob, 2014). NCAM1 and NCAM2 are structurally similar, being composed of five N-terminal Ig domains (IgI-V) and two fibronectin type III (Fn3) repeats (Fn3I-II) (Figure 3A). Alternative splicing generates three major NCAM1 isoforms, which have identical extracellular domains and differ in the membrane attachment. Two longer isoforms of NCAM1, designated NCAM140 and NCAM180 according to their molecular weight, are transmembrane proteins with a longer intracellular tail in NCAM180. The shortest NCAM1 isoform, designated NCAM120, is a glycosylphosphatidylinositol (GPI)-anchored protein lacking the intracellular domain. Two major NCAM2 isoforms also have identical extracellular domains. The longer NCAM2 isoform is a transmembrane protein, whereas the shorter isoform is GPI-anchored to the plasma membrane. NCAMs are expressed in many tissues but are particularly enriched in the brain, where they participate in the regulation of neurite outgrowth, synaptogenesis and synaptic plasticity (Sytnyk et al., 2017; Rasmussen et al., 2018).

##### NCAMs interact with APP

NCAM1 forms a complex with APP in the mouse brain, with the E2 domain of APP and the extracellular domain of NCAM1 mediating this interaction (Chen and Dou, 2012) (Figure 3Ai). NCAM180, a splice variant of NCAM1 with a longer cytoplasmic tail, does not, however, associate with APP, suggesting that the APP/NCAM1 complex formation may also be dependent on an additional intracellular mechanism. The interaction of APP with NCAM1 is conserved in Drosophila melanogaster, where the APP homolog, APPL, binds to the NCAM ortholog Fasciclin 2 (Ashley et al., 2005; Mao and Freeman, 2009). Human APP was also found in a complex with NCAM2 in the brain of transgenic APP23 mice (Leshchyns’ka et al., 2015).

##### Role of APP in regulating NCAM1-dependent neurite outgrowth and synaptogenesis

APP and NCAM1 trigger the mitogen-activated protein kinase (MAPK) pathway *via* phosphorylation of ERK1 and ERK2. Co-expression of NCAM1 and APP in COS7 cells promotes phosphorylation of ERK1,2 to a larger extent than found in cells expressing either protein alone (Chen and Dou, 2012) (Figure 3Ai,ii). This synergistic effect requires the extracellular, but not the intracellular domain of APP. Both APP and NCAM1 independently promote neurite outgrowth in mouse hippocampal neurons, and when present together they increase neurite outgrowth synergistically (Chen et al., 2016). The fact that the latter effect can be induced by secreted sAPPα is consistent with a non-essential role of the intracellular domain of APP, altogether indicative of a ligand-receptor interaction between APP and NCAM1 (Figure 3Aii).

The role of the interaction between NCAM and APP in synaptogenesis was shown in Drosophila, where Fasciclin 2 promotes synapse formation by interacting with APPL, which initiates signaling *via* the adaptor protein Mint1 (Ashley et al., 2005).

##### NCAMs in AD

In AD, NCAM1 levels are reduced in the frontal and temporal cortex, while levels of proteolytic NCAM1 fragments in the serum are increased (Todaro et al., 2004; Aisa et al., 2010). The levels of NCAM1 carrying polysialic acid, an unusual carbohydrate predominantly found on NCAM1, are similarly diminished in the entorhinal cortex in AD-affected brains inversely correlating with hyperphosphorylated tau load (Murray et al., 2016). The levels of polysialylated NCAM1 are however increased in the AD hippocampus (Mikkonen et al., 1999).

NCAM2 is enriched in the human hippocampus, a brain region highly susceptible to Aβ-induced toxicity. In brains of people with AD, NCAM2 levels are reduced in hippocampal synapses and the levels of soluble NCAM2 are elevated suggesting increased shedding of NCAM2 from synaptic membranes (Leshchyns’ka et al., 2015). The overall levels of NCAM2 and the levels of its phosphorylation are however increased in AD brains (Leshchyns’ka et al., 2015; Sathe et al., 2020). Together these data suggest that NCAMs play a role in AD.

##### NCAMs regulate the amyloidogenic processing of APP

Co-expression of NCAM1 with APP in CHO cells reduces production of both Aβ_40_ and Aβ_42_, suggesting that NCAM1 modulates APP processing (Chen and Dou, 2012) (Figure 3Ai). The mechanism underlying this effect is currently unknown.

A single nucleotide polymorphism (SNP) in *NCAM2* (rs2212624) is associated with the development of late-onset AD (Kimura et al., 2006), while another SNP in *NCAM2* (rs1022442) is associated with high Aβ levels in the cerebrospinal fluid (CSF) (Han et al., 2010), together suggesting that NCAM2 may be implicated in Aβ-dependent AD pathology. The association between SNPs in *NCAM2* and the levels of Aβ in CSF (Han et al., 2010) suggests that NCAM2 may influence Aβ production, presumably by interacting with APP.

##### Role of NCAMs in Aβ toxicity

A peptide derived from the fibroblast growth factor receptor (FGFR)-binding region of NCAM1 within the Fn3II domain prevents neurodegeneration and cognitive impairment in AβO-treated rats (Klementiev et al., 2007). The peptide mimics NCAM1 functions by binding to and activating FGFR (Neiiendam et al., 2004). Together these data suggest that the loss of NCAM1-FGFR interactions and attenuation of NCAM1-dependent signaling contribute to Aβ toxicity.

NCAM2 interacts with AβOs in brains of transgenic APP23 mice, and AβOs increase shedding of synaptic NCAM2 in cultured hippocampal neurons, compromising synaptic adhesion and inducing synapse disassembly (Leshchyns’ka et al., 2015) (Figure 3Aiii).

#### L1 family

The L1 family is a group of IgSF CAMs that includes L1, close homologue of L1 (CHL1), neurofascin and NgCAM-related CAM (NrCAM). L1 is a transmembrane protein with a short cytoplasmic tail and extracellular domain composed of six Ig-like domains and five Fn3 repeats (Figure 3B). L1 is expressed in a variety of tissues, but is enriched in the nervous system, where it plays a role in synaptogenesis, neurite outgrowth, and neuromuscular junction stability (Sytnyk et al., 2017).

##### Role of APP in regulating L1 family functions

APP interacts with neuronal-glial CAM (NgCAM), the chicken ortholog of L1, in the chick brain, and APP and sAPPα enhance NgCAM-dependent axon growth in retinal ganglion cells (Osterfield et al., 2008) (Figure 3Bi). In the mouse brain, APP interacts with another L1 family member, neurofascin (Bai et al., 2008). The functional role of this interaction remains to be investigated.

##### L1 family members in AD and their role in the amyloidogenic processing of APP and Aβ toxicity

In the CSF of AD patients, the levels of L1 proteolytic fragments are increased (Strekalova et al., 2006), while neurofascin levels are reduced (Brinkmalm et al., 2018).

The levels of full-length APP and αCTF are elevated in HEK293T cells co-expressing NgCAM, suggesting a role for the latter in modulation of APP stability and/or processing (Osterfield et al., 2008) (Figure 3Bi).

Aβ_42_ peptides bind to L1 *in vitro via* its second Fn3 domain (Djogo et al., 2013) (Figure 3Bii). This interaction reduces the formation of high-molecular weight (MW) forms of AβOs, with a corresponding increase in levels of Aβ monomers, trimers, and tetramers. Hippocampal L1 levels are reduced in aged APPswe mice, a mouse model of AD overexpressing human APP with a Swedish (KM670/671NL) mutation, which exhibits Aβ deposition with age (Hu et al., 2022). In APP/PS1 mice, a mouse AD model co-expressing mutated human APP with a Swedish mutation and mutated presenilin 1, overexpression of L1 using adeno-associated viruses results in a reduced Aβ plaque load and Aβ_42/40_ ratio, as well as milder hippocampal synapse loss and astrogliosis (Djogo et al., 2013). A 70 kDa fragment of L1 (L1-70) generated by proteolysis of L1 by serine proteases also reduces Aβ load in mice. This occurs *via* translocation of L1-70 to the nucleus inducing cytokine expression and the clearance of Aβ plaques by activated microglia (Hu et al., 2022). While microglial activation is important for Aβ clearance, in prolonged or extreme form it results in inflammation exacerbating neuronal damage in AD (Heneka et al., 2015).

Together, these findings allude to a protective role for L1 in AD *via* the prevention of aggregation and promotion of Aβ clearance.

#### Contactins

Contactins are a family of CAMs within the IgSF comprising six members, including contactin-1 (also designated F3), contactin-2 (also named TAG1 or TAX1), contactin-3 (also named BIG-1 or PANG), contactin-4 (also designated BIG-2), contactin-5 (also named NB-2), and contactin-6 (also designated NB-3). Contactins are composed of six Ig domains and four Fn3 domains attached to plasma membrane *via* a GPI anchor (Figure 3C). They are primarily expressed in the brain where they accumulate in axons, contributing to control of axon growth and guidance, as well as to other functions distinct for each family member (Shimoda and Watanabe, 2009; Gennarini et al., 2017; Chatterjee et al., 2019).

##### Role of APP in regulating contactin family functions

In the mouse brain, APP was shown to interact with contactin-1 (Bai et al., 2008; Puzzo et al., 2015), contactin-2 (Ma et al., 2008), and contactin-4 (Osterhout et al., 2015). Contactin-3 and -4 were demonstrated to interact with APP in the chick brain (Osterfield et al., 2008). *In vitro* assays show that contactin-3 and -4 bind to APP with the highest affinity amongst the contactin family *via* a conserved interaction interface between the E1 CuBD of APP and second Fn3 domain of Contactin-3 and -4 (Peng et al., 2019; Karuppan et al., 2022) (Figure 3C). Contactin-5 also binds to APP *via* this interface, suggesting the binding site is likely to be similar for all contactins (Karuppan et al., 2022). Contactin-1 and -2 show little to no binding to APP in these *in vitro* assays, suggesting additional factors may mediate such interactions in the brain. Contactin-6 does not bind to APP *in vitro*, and this interaction has not thus far been identified *in vivo*, potentially making contactin-6 unique within the contactin family.

The interaction of contactin-4 with APP is required for axon-target matching in mouse retinal ganglion cells, regulating the circuitry involved in vision stabilization (Osterhout et al., 2015) (Figure 3C). The physiological role of the interactions between other contactins and APP is yet to be determined.

##### Contactin family members in AD and their role in the amyloidogenic processing of APP and Aβ toxicity

In mice, contactin-1 expression in the hippocampus decreases with age being associated with age-dependent cognitive decline (Shimazaki et al., 1998; Puzzo et al., 2015). Single nucleotide polymorphisms in the contactin-2 coding gene are associated with late-onset AD and contactin-2 CSF levels are reduced in AD and correlate with Aβ_40_ and hyperphosphorylated tau levels (Medway et al., 2010; Chatterjee et al., 2018). The levels of contactin-2 also decrease with age in mice (Tachi et al., 2010). The chromosomal region encompassing the contactin-4 coding gene was found to have suggestive linkage to late-onset AD (Blacker et al., 2003; Bamford et al., 2020).

Contactin-1 is suggested to modulate the α-/β-cleavage of APP as aged transgenic mice overexpressing contactin-1 display reduced sAPPβ and increased sAPPα levels compared to similarly aged wild-type mice (Puzzo et al., 2015) (Figure 3C). The levels of Aβ_42_ are decreased, while the levels of Aβ_40_ remain unchanged in these transgenic mice, suggesting that the preferred γ-cleavage site may also be influenced by contactin-1 overexpression. Contactin-2 may similarly influence the α-/β-cleavage of APP as mouse embryonic fibroblasts overexpressing contactin-2 show increased production of both α- and β-CTFs, with a more prominent rise in α-CTF levels (Ma et al., 2008). However, contactin-2 does not affect sAPPα or sAPPβ levels in HEK293 cells or cultured cortical neurons (Rice et al., 2013). Contactin-2 promotes the γ-cleavage of APP-CTFs as demonstrated by a decrease in AICD release in *CNTN2−/−* embryonic mouse brains (Ma et al., 2008). This contactin-2-dependent γ-cleavage and release of AICD initiates a signaling pathway that inhibits neurogenesis (Figure 3C). Contactin-2 is also suggested to suppress neuronal cell death, as it competitively inhibits the binding of transforming growth factor β2 (TGFβ2) to APP (Tachi et al., 2010), an interaction known to induce neuronal apoptosis (Hashimoto et al., 2005). In HEK293 cells, co-expression of contactin-4 increases the levels of full-length APP and αCTF suggesting that contactin-4 also regulates the processing of APP (Osterfield et al., 2008) (Figure 3C).

Together, these data suggest that at least some members of the contactin family regulate the function and processing of APP, and that the age-related reduction in contactin expression may contribute to the shift towards amyloidogenic processing that leads to AD.

#### Other IgSF CAMs

The small GPI-anchored CAM Thy-1 has also been identified as a binding partner of APP (Bai et al., 2008), while neurotrimin and opioid-binding protein/CAM have been found to bind AβF (Verdier et al., 2005). The role these interactions play in normal physiology and amyloid-dependent pathology requires further investigation.

### Integrins

**FIGURE 4:**
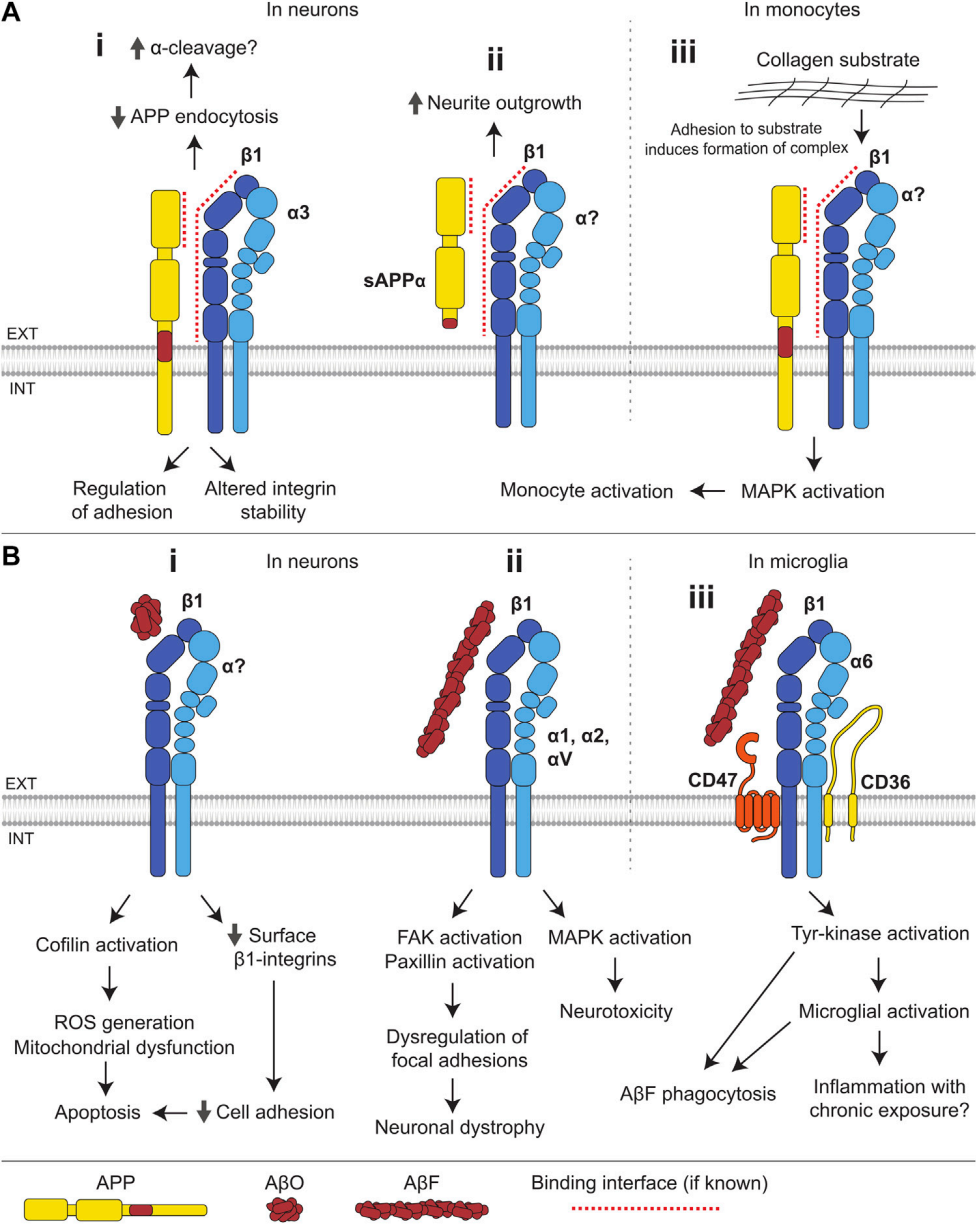
Interactions of integrins with APP and Aβ. **(A) (i)** The E1 domain of APP interacts with β1-and α3-integrins. Integrins suppress endocytosis of APP and thereby may facilitate its α-cleavage. APP influences integrin stability and integrin-dependent adhesion. **(ii)** In neurons, sAPPα binds to β1-integrins and induces neurite outgrowth. **(iii)** In monocytes, adhesion to a collagen substrate induces the formation of an APP-β1-integrin complex, which activates MAPK signaling resulting in monocyte activation. **(B) (i)** In neurons, binding of AβOs to β1-integrins leads to a reduction in the cell surface levels of β1-integrins and compromised cell adhesion, and results in activation of the f-actin severing protein, cofilin, mitochondrial dysfunction and ROS generation, leading to apoptosis. **(ii)** AβFs bind several integrin subunits (β1, α1, α2, and αV) and induce MAPK activation and neurotoxicity. Binding of AβF to β1-integrin also induces FAK and paxillin activation, leading to dysregulation of integrin-mediated focal adhesion and contributing to neuronal dystrophy. **(iii)** In microglia, AβFs bind to a receptor complex composed of α6β1-integrin, CD47, and CD36, and induce tyrosine-kinase activation, stimulating AβF phagocytosis and microglial activation.

Integrins form a large and diverse family of ubiquitously expressed transmembrane CAMs (Barczyk et al., 2009). They are heterodimers composed of α and β subunits, combining to form at least 24 different pairs with distinct functions and expression patterns (Takada et al., 2007) (Figure 4). Integrins are the primary mediators of cell-to-ECM adhesion throughout the body and serve a wide variety of functions including regulation of cellular growth and migration (Howe et al., 1998; Huttenlocher and Horwitz, 2011). Integrins are widely expressed in cells of the nervous system, where they play important roles in neurite outgrowth, synaptic plasticity, and neural immune function (Archelos et al., 1999; Clegg et al., 2003).

#### Integrins interact with APP

In the mouse and rat brain, APP binds to β1-integrins and α3-integrins, but not to αM-integrins (Young-Pearse et al., 2008; Hoe et al., 2009) (Figure 4A). APP colocalizes strongly with α1β1-and α5β1-integrins in cultured neurons, and with α1β1- but not α5β1-integrins in cultured astrocytes (Yamazaki et al., 1997), suggesting cell type-specific interactions between APP and different integrin subunits. The β1-integrin-APP complex is formed *via* the interaction of the E1 domain of APP with the extracellular domain of β1-integrin (Young-Pearse et al., 2008). Binding sites within APP for other integrin subunits remain unknown. APP and β1-integrins may also be linked by cytoplasmic adaptor proteins, such as Dab1 and Fe65, which both APP and β1-integrins bind to (Young-Pearse et al., 2008).

#### Role of APP in regulating integrin-dependent functions

Studies in multiple cell types suggest that APP regulates integrin-mediated adhesion, a function consistent with the ubiquitous expression of both proteins. The loss of APP in endothelial cells reduces expression of β1-and β3-integrins, compromising attachment of cells to collagen and fibronectin substrates (Ristori et al., 2020). Adhesion of THP-1 monocytes to collagen substrates is also dependent on APP expression, wherein adhesion induces the formation of a receptor complex containing β1-integrins and APP, initiating MAPK signaling and facilitating monocyte activation (Sondag and Combs, 2004) (Figure 4Aiii). In contrast, the loss of APP in mice leads to an increase in the levels of β1-and α3-integrins in the brain (Hoe et al., 2009), suggesting that the integrin-APP interactions in cells of the nervous system may differ from those in other tissues. In cultured neurons, blockade of β1-integrins with anti-β1-integrin antibodies leads to inhibition of neurite outgrowth induced by sAPPα, suggesting that β1-integrins function as receptors for soluble forms of APP (Young-Pearse et al., 2008) (Figure 4Aii).

#### β1-integrins regulate the processing of APP

In cultured hippocampal neurons overexpressing β1-integrin and APP, APP internalization is reduced and APP levels at the cell surface are increased (Hoe et al., 2009), suggesting that β1-integrins facilitate the α-cleavage of APP which is known to occur predominantly at the cell surface (Parvathy et al., 1999) (Figure 4Ai). In agreement, overexpression of β1-integrins in HEK293 cells endogenously expressing APP leads to an increase in sAPPα production, however, this effect could not be reproduced in cultured cortical neurons (Rice et al., 2013). The levels of β1-integrins are reduced in the brain of the Tg2576 mouse model of AD overexpressing a mutant form of APP (isoform 695) with the Swedish mutation (KM670/671NL). This model displays elevated Aβ levels and ultimately amyloid plaques (Hoe et al., 2009), supporting the idea that low integrin levels correlate with increased amyloidogenic APP processing. Integrin-mediated signaling was also implicated in the control of APP processing in several genome-wide siRNA screens (Camargo et al., 2015; Chapuis et al., 2017). β1-integrins interact with a 109 amino acid APP-CTF (Ghiso et al., 1992). They may therefore bind to α- and β-CTFs of APP and influence their γ-cleavage.

#### Role of integrins in Aβ toxicity

Integrins bind to Aβ monomers, oligomers, and fibrils. The RHDS sequence located near the N-terminus of Aβ is similar to the known integrin recognition sequence RGDS (Ghiso et al., 1992). CHO cells expressing α5β1-integrins attach to non-fibrillar Aβ-coated surfaces, and this attachment is blocked by soluble RGDS sequence-containing peptides or anti-α5-integrin antibodies (Matter et al., 1998). The binding of integrins to Aβ is subunit specific because CHO cells expressing αvβ1-integrin, but not αvβ3-integrin, also attach to Aβ-coated surfaces. Aβ_40_ peptides also bind to αIIbβ3-integrin (platelet integrin) *via* the RHDS sequence, however, other sequences in Aβ_40_ are also involved (Donner et al., 2016; Donner et al., 2018). The binding of α6-integrins and β1-integrins to AβF is mediated by an epitope distinct from the RHDS sequence (Bamberger et al., 2003; Venkatasubramaniam et al., 2014). β1-integrin also binds AβOs, and β1-integrin conditional knock-out mice demonstrate reduced binding of AβOs to neurons (Woo et al., 2015).

The binding of AβOs to β1-integrins in neurons results in the activation of cofilin, an f-actin severing protein, leading to the depletion of f-actin, mitochondrial dysfunction, ROS generation, and apoptosis (Woo et al., 2015) (Figure 4Bi). β1-integrin also mediates a transient increase in spine density and dendritic complexity following AβO treatment (Ortiz-Sanz et al., 2020). Changes in function of oligodendrocytes, the myelinating glial cells of the central nervous system, are associated with the onset of neurodegeneration in AD. It is noteworthy that the binding of AβOs to β1-integrin in oligodendrocytes leads to the activation of protein tyrosine kinase Fyn and serine/threonine-specific Ca^2+^/calmodulin-dependent protein kinase II (CaMKII), promoting differentiation, maturation, and survival of these cells (Quintela-López et al., 2019). This may represent a physiological role of Aβ which may be lost in favor of toxicity with chronic Aβ exposure.

Binding of AβF to β1-integrin in neurons results in activation of focal adhesion kinase (FAK) and the focal adhesion scaffolding protein, paxillin, leading to the formation of aberrant focal adhesion-like structures (Grace and Busciglio, 2003; Han et al., 2013). AβF-induced neuronal dystrophy is dependent on β1-integrin-induced paxillin activation, indicating that dysregulation of focal adhesions may be central to Aβ toxicity (Figure 4Bii). Furthermore, AβF-induced neurotoxicity appears to be driven by the MAPK pathway, dependent on specific integrins, with neurotoxicity mediated by α1-integrin in hippocampal neurons and α2-, αV, and β1-integrins in cortical neurons (Anderson and Ferreira, 2004; Wright et al., 2007). Binding of AβF to a receptor complex composed of α6β1-integrin, IgSF CAM CD47, and B-class scavenger receptor CD36 in microglia, immune effector cells of the central nervous system, causes Fyn activation, triggering microglial cell activation and leading to a potentially deleterious inflammatory response (Bamberger et al., 2003) (Figure 4Biii). Tyrosine kinase-dependent signaling *via* this receptor complex induces phagocytosis of AβF, which may facilitate Aβ clearance (Koenigsknecht and Landreth, 2004). Blockade of β1-integrins prevents AβF-induced tyrosine kinase signaling, ROS generation, and interleukin-1β production in THP-1 monocytes (Bamberger et al., 2003). AβF also stimulates histamine secretion from mast cells *via* binding to a β1-integrin-CD47 receptor complex (Niederhoffer et al., 2009).

While aberrant overactivation of some integrin signaling pathways appears to be a common response to Aβ, other integrin-dependent pathways are silenced in AD mouse models. For example, integrin-linked kinase (ILK) levels and activity are reduced in APP/PS1 mice expressing a chimeric mouse/human APP with a Swedish mutation and a mutant human presenilin 1 in the central nervous system neurons. Overexpression of ILK rescues hippocampal neurogenesis and memory deficits in this AD mouse model (Xu et al., 2018).

Integrin-mediated cell-to-cell and cell-to-ECM adhesion also appears to be a target of Aβ toxicity, as Aβ peptides partially block β1-integrin-mediated adhesion of SH-SY5Y cells to a fibronectin substrate (Sabo et al., 1995). AβOs induce the loss of cell surface β1-integrins in cultured neurons and α1β1-integrins in neuroblastoma cell lines (Bozzo et al., 2004; Bozzo et al., 2010; Woo et al., 2015). Overexpression of α5β1-integrins in human neuroblastoma IMR-32 cells results in inhibition of Aβ-induced apoptosis (Matter et al., 1998), suggesting that the loss of integrins contributes to AβO-induced neuronal death in AD (Figure 4Bi).

### Cadherins

**FIGURE 5:**
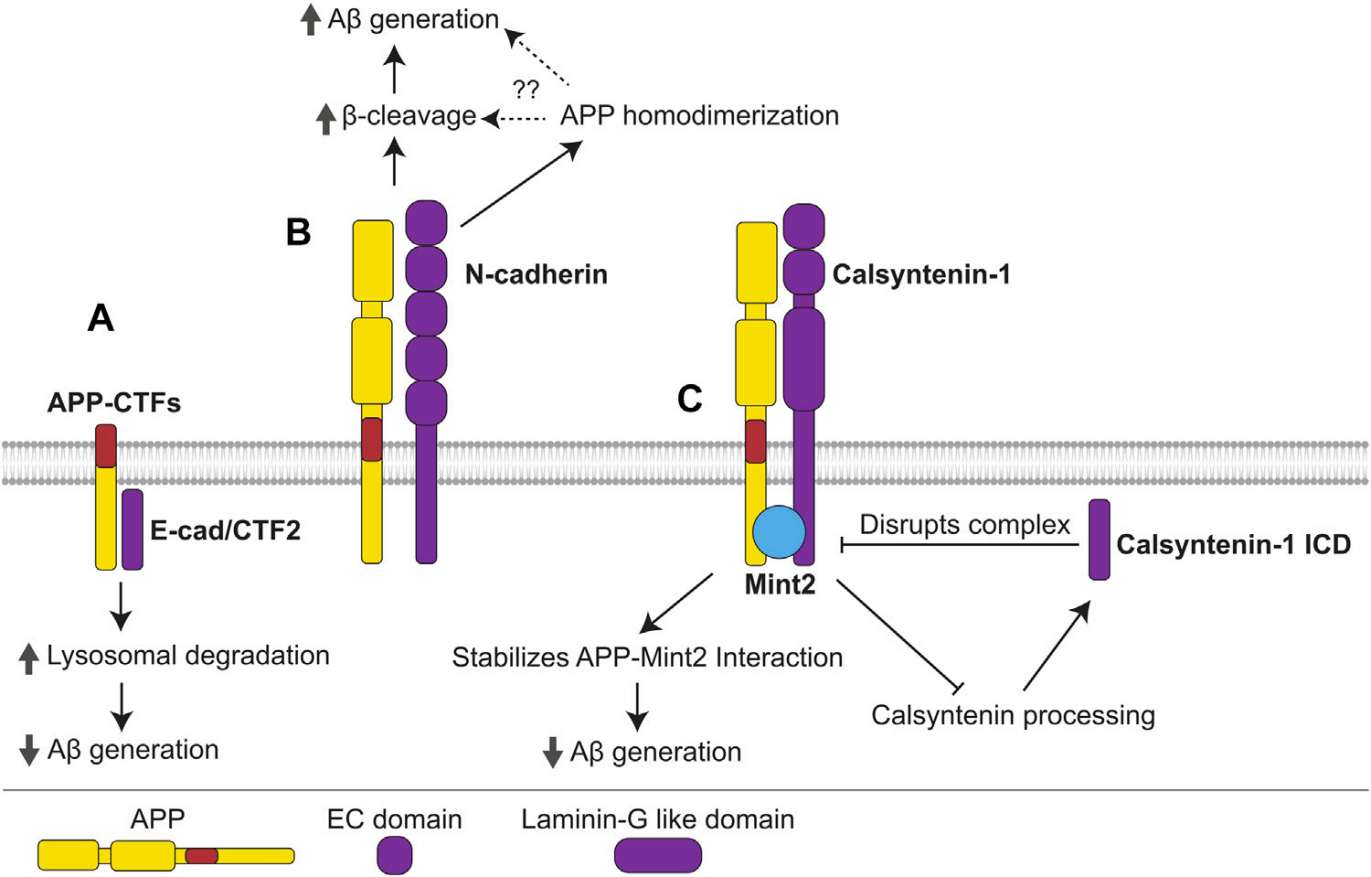
Interactions of cadherins with APP. **(A)** The γ-cleavage product of E-cadherin, E-cad/CTF2, interacts with APP-CTFs, promoting their lysosomal degradation and precluding Aβ generation. **(B)** N-cadherin interacts with APP promoting its homodimerization, β-cleavage, and Aβ generation. N-cadherin-induced APP homodimerization may promote β-cleavage and Aβ generation, however, this mechanism needs to be confirmed. **(C)** APP forms a complex with the non-classical cadherin, calsyntenin-1, and adaptor protein Mint2. The APP-Mint2 interaction, which may suppress Aβ generation, is stabilized in the tripartite complex with calsyntenin-1, thus enabling greater suppression of Aβ generation. Formation of the APP-Mint2-calsyntenin-1 complex also precludes calsyntenin-1 processing, reducing generation of the γ-cleavage product, calsyntenin-1 ICD, which disrupts the APP-Mint2-calsyntenin-1 complex.

Cadherins are a family of widely expressed CAMs characterized by the presence of the extracellular cadherin (EC) domains and mediating calcium-dependent homophilic adhesion between cells. Classical cadherins such as E- or N-cadherin are transmembrane glycoproteins with a short cytoplasmic tail and extracellular domain composed of five EC domains (Figure 5B). Other cadherins contain variable numbers of EC and other domains. Cadherins play various roles in regulating cell migration, cytoskeleton organization, and are crucial components of adherens junctions in epithelia and endothelia (Angst et al., 2001; Halbleib and Nelson, 2006; Kowalczyk and Nanes, 2012; Shih and Yamada, 2012). In the brain, cadherins regulate neurite outgrowth and the formation of synaptic contacts, and are important for overall neural development (Zhu and Luo, 2004; Arikkath and Reichardt, 2008; Hansen et al., 2008).

#### Cadherins in AD and their role in the amyloidogenic processing of APP

The levels of N-cadherin proteolytic fragments are increased in the CSF and plasma of AD patients (Choi et al., 2020), while N-cadherin expression is upregulated in the cerebral cortex of Aβ aggregate-injected mice (Kong et al., 2005), suggesting deregulation of N-cadherin expression or cleavage of this glycoprotein in AD.

In the mouse brain, N-cadherin interacts with APP and enhances APP homodimerization as well as production of sAPPβ and Aβ (Asada-Utsugi et al., 2011) (Figure 5B). Similarly to APP, E-cadherin is processed by ADAM10 and γ-secretase (Marambaud et al., 2002; Maretzky et al., 2005). The cytoplasmic fragment of E-cadherin, termed E-cad/CTF2, which is released following γ-secretase cleavage of E-cadherin, interacts with APP-CTFs in CHO cells, promoting their lysosomal degradation and inhibiting the production of Aβ and AICD (Agiostratidou et al., 2006) (Figure 5A). E-cadherin was identified as an interacting partner of APP *in silico* using a protein-protein interaction tool (Dursun and Gezen-Ak, 2017). This approach also identified β-catenin, which is a cytoplasmic adaptor protein that links E-cadherin to the cytoskeleton (Tian et al., 2011), suggesting that APP modulates cadherin functions in cytoskeletal organization, signaling, and adherens junction maintenance.

In the mouse brain, APP interacts with the non-classical cadherins calsyntenin-1 and -3 (Bai et al., 2008). These cell adhesion molecules are highly expressed in neurons and accumulate in the post-synaptic membrane (Hintsch et al., 2002). Calsyntenin-1 forms a complex with APP and Mint2, stabilizing the APP-Mint2 interaction, suppressing amyloidogenic processing and reducing Aβ production (Araki et al., 2003; Gotoh et al., 2020) (Figure 5C). Accordingly, calsyntenin-1 deficiency in APP23 mice results in increased plaque deposition. On the other hand, the intracellular domain of calsyntenin-1 released following γ-secretase cleavage (calsyntenin-1 ICD) disrupts the APP-Mint2-Calsyntenin-1 complex, promoting Aβ generation (Takei et al., 2015). Since the tripartite complex additionally protects calsyntenin-1 from cleavage (Araki et al., 2004), calsyntenin-1 ICD production may promote further calsyntenin-1 cleavage and ICD production, leading to a positive feedback loop that generates increasing amounts of Aβ (Figure 5C).

### Neurexins and neuroligins

**FIGURE 6:**
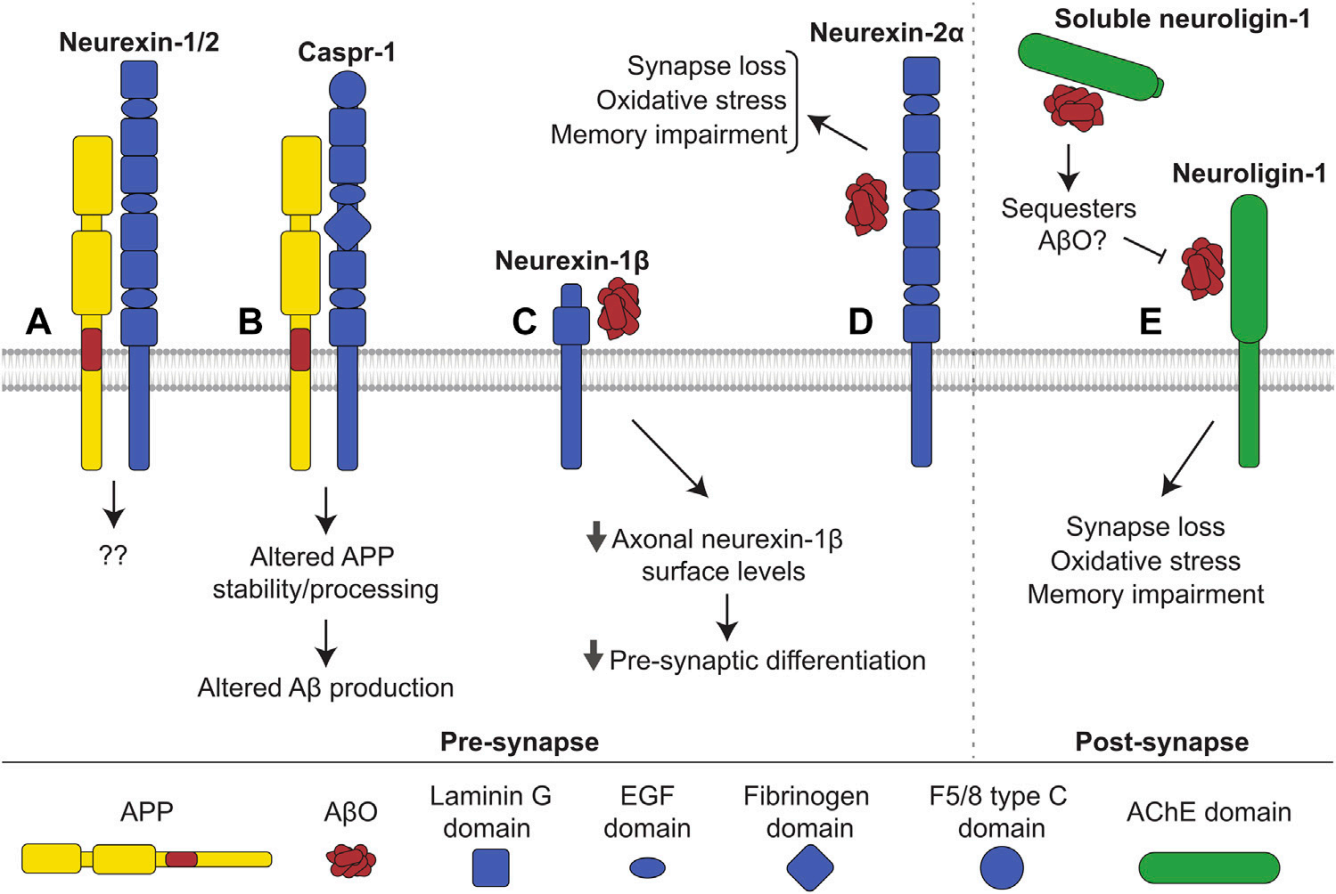
Interactions of neurexin and neuroligin with APP and Aβ. **(A)** Neurexin-1 and -2 interact with APP in mouse brains, with unknown consequence. **(B)** Caspr-1 interacts with APP. This interaction alters stability and processing of APP influencing production of Aβ. It is uncertain whether the interaction increases or decreases amyloidogenic processing. **(C)** AβOs bind to neurexin-1β, reducing its levels at the axonal cell surface and hindering presynaptic differentiation. **(D)** AβOs additionally bind to neurexin-2α triggering oxidative stress, synapse loss, and memory impairments in mice. Similarly, **(E)** AβOs interact with neuroligin-1 at the post-synapse, inducing oxidative stress, synapse loss, and memory impairments in mice. Shedding releases soluble neuroligin-1 which also binds to and likely sequesters AβOs, preventing interactions with membrane-bound neuroligin-1 and other receptors.

Neurexins are a family of transmembrane CAMs, which includes three classical neurexins (neurexin-1 to -3) and five contactin-associated proteins (Caspr-1 to -5) named for their close association with contactins. Each neurexin gene encodes an α and β isoform, which differ in their extracellular part. The extracellular domains of α-neurexins are composed of six laminin G-like domains and three EGF-like regions, while the extracellular domains of β isoforms contain only a single laminin G-like repeat (Reissner et al., 2013). The extracellular domains of contactin-associated proteins contain four laminin G-like domains, two EGF-like regions, an F5/8 type C domain, and a fibrinogen-like part (Figure 6B).

Neuroligins are a family of transmembrane CAMs, which includes five members (neuroligin-1 to -3, 4X and 4Y). The extracellular domains of neuroligins mostly consist of an acetylcholinesterase (AChE)-homologous domain, which binds in a calcium-dependent manner to the laminin G-like domains of neurexins (Bemben et al., 2015). The interaction between presynaptic neurexins and postsynaptic neuroligins plays an important role in synapse formation and maturation being critical for overall neural development (Craig and Kang, 2007; Bang and Owczarek, 2013).

#### Role of neurexin family members in the amyloidogenic processing of APP

Cytosolic domains of neurexins and APP interact with Mint1 and Mint2 (Biederer and Südhof, 2000), and neurexin-1 and -2 were found in a complex with APP in the brains of transgenic mice expressing affinity tagged APP (Norstrom et al., 2010) (Figure 6A). APP also interacts with Caspr-1 in the mouse brain (Fan et al., 2013) (Figure 6B). Caspr-1 interacts with and is cleaved by γ-secretase, and its silencing results in a drastic reduction of Aβ production in HEK293 cells (Hur et al., 2012). The levels of Caspr-1 are increased in APP/PS1 mice (Fan et al., 2013). Caspr-1 loss of function leads to a decrease in sAPPα production in brain endothelial cells *via* transcriptional regulation of the secondary α-secretase ADAM9 (Tang et al., 2020). Interestingly, Caspr-1 overexpression results in reduced levels of APP, APP-CTFs and Aβ in CHO cells transfected with APP containing an Indiana (V717F) mutation (Fan et al., 2013), which affects binding of APP to γ-secretase (De Jonghe et al., 2001).

#### Role of neurexin family members in Aβ toxicity

AβOs bind to neurexin-1, -2, and -3 in transfected COS7 cells (Naito et al., 2017). The binding of AβOs to β-neurexins has no effect on neurexin-neuroligin interactions but hinders the neurexin-mediated excitatory presynaptic differentiation observed when hippocampal neurons were co-cultured together with neuroligin-expressing HEK293 cells. This occurs *via* a reduction in neurexin-1β levels at the axonal cell surface induced by AβOs (Figure 6C). In accordance, β-neurexins, but not α-neurexins, are downregulated in synapses of the J20 APP mouse model of AD, which overexpresses human APP with the Swedish and Indiana mutations linked to familial AD (Naito et al., 2017). AβOs also bind neurexin-2α in human brain tissue (Brito-Moreira et al., 2017) (Figure 6D). Blockade of the neurexin-2α-AβO interaction using antibodies against neurexin-2α reduces the binding of AβOs to cultured hippocampal neurons and prevents AβO-induced oxidative stress and synapse loss. AβO-induced memory impairment in mice is attenuated after injection of the aforementioned anti-neurexin-2α antibodies (Brito-Moreira et al., 2017). Neurexins-1, -2, and -3 have been reported to bind to AβF in CSF of AD patients (Rahman et al., 2018).

#### Role of neuroligins in Aβ toxicity

AβOs bind to neuroligin-1 in human brain tissue and in the rat brain (Dinamarca et al., 2015; Brito-Moreira et al., 2017). Blockade of the neuroligin-1-AβO interaction reduces binding of AβOs to neurons, inhibits AβO-induced oxidative stress and synapse loss in cultured neurons, and prevents memory impairments in mice (Brito-Moreira et al., 2017). Soluble neuroligin-1 binds to AβOs and reduces excitatory synaptotoxicity (Dinamarca et al., 2015), most likely by sequestering AβOs and inhibiting their interaction with membrane-bound neuroligin-1 (Figure 6E). On the other hand, neuroligin-1 deficient neurons are more vulnerable to AβO-induced toxicity, and AβO-induced impairments in learning are more severe in neuroligin-1 deficient mice (Dufort-Gervais et al., 2020). Neuroligin-1 levels are also reduced in the hippocampus of people with AD (Dufort-Gervais et al., 2020). These seemingly contradictory findings may suggest that although neuroligin-1 mediates toxicity *via* its interaction with AβOs, loss of neuroligin-1 and its associated physiological functions may weaken synapses and increase vulnerability to AβO toxicity through other receptors. AβOs also bind to neuroligin-2, but not neuroligin-3 (Brito-Moreira et al., 2017). In contrast to neuroligin-1, neuroligin-2 is localized to inhibitory synapses (Varoqueaux et al., 2004), and the effects of its interaction with AβOs remain unknown.

### Prion protein

The cellular prion protein (PrP^c^) is a small glycoprotein well-known for its role in prion diseases such as Creutzfeldt-Jakob disease, where it exists in a misfolded and aggregated form termed prion protein scrapie (PrP^Sc^) (Atkinson et al., 2016). PrP^c^ consists of a disordered N-terminal domain and C-terminal α-helical region attached to the membrane *via* a GPI anchor (Watts et al., 2018). PrP^c^ is expressed in a variety of tissues but is particularly enriched in the brain where is plays a role in regulating cell adhesion, neuronal development, synaptic plasticity, and myelin maintenance (Roucou and LeBlanc, 2005; Petit et al., 2013; Wulf et al., 2017; Watts et al., 2018).

#### Role of APP in regulating PrP^c^-dependent functions

**FIGURE 7:**
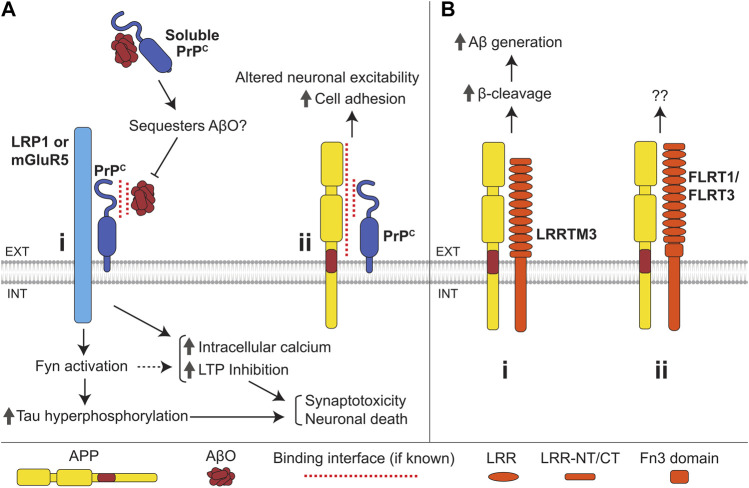
Interactions of the cellular prion protein (PrP^c^) and LRR superfamily CAMs with APP and Aβ. **(A) (i)** Binding of AβOs to PrP^c^ induces an increase in intracellular Ca^2+^ levels, LTP inhibition, Fyn activation, and tau hyperphosphorylation, ultimately resulting in synaptotoxicity and neuronal death. PrP^c^ is anchored to the outer leaflet of the plasma membrane and transmits the AβO-induced signals across the plasma membrane by interacting with the transmembrane proteins LRP1 and mGluR5. Soluble PrP^c^ binds to and can sequester AβOs, preventing membrane-bound PrP^c^-mediated AβO toxicity. **(ii)** Interaction of APP with the N-terminal domain of PrP^c^ enhances cell adhesion *via* an unknown mechanism and may also regulate neuronal excitability. **(B) (i)** LRRTM3 interacts with APP promoting its β-cleavage and facilitating Aβ generation. **(ii)** APP also interacts with FLRT1 and FLRT3, with unknown consequence.

PrP^c^ interacts with APP in the mouse brain, zebrafish, and N2a cells (Schmitt-Ulms et al., 2004; Bai et al., 2008; Kaiser et al., 2012) (Figure 7Aii). APP was pulled down from human brain lysate with the N-terminal domain of PrP^c^ used as bait (Ulbrich et al., 2018). In zebrafish embryos, knockdown of either APP or PrP^c^ homologs was shown to have no effect on cell aggregation, however, knockdown of both significantly reduced the propensity for aggregation. (Kaiser et al., 2012). The loss of APP in zebrafish results in increased seizures upon exposure to low doses of convulsant, and this effect is lost in fish lacking PrP^c^ suggesting that APP regulates neuronal excitability in a PrP^c^-dependent manner (Kanyo et al., 2020).

#### PrP^c^ in AD and its role in the amyloidogenic processing of APP

There are some similarities between prion diseases and AD, and the gene encoding for PrP^c^ (*Prpn*) has been identified as a potential susceptibility gene for AD (Riemenschneider et al., 2004; Bertram et al., 2007). PrP^c^ levels are reduced in the temporal cortex of patients suffering from sporadic AD, and this reduction correlates with increased clinical severity of the disease (Whitehouse et al., 2013).

PrP^c^ modulates APP processing by interacting with BACE1, where PrP^c^ binds to immature BACE1 in the Golgi, preventing its export and maturation (Parkin et al., 2007; Griffiths et al., 2011). PrP^c^ reduces Aβ levels in mouse brains, and PrP^c^ levels inversely correlate with Aβ load in AD (Whitehouse et al., 2013). It is unknown whether PrP^c^ also controls APP processing by directly interacting with APP.

#### Role of PrP^c^ in Aβ toxicity

AβOs bind to the N-terminal region of PrP^c^ in AD brains but not in brains of non-demented controls (Chen et al., 2010; Dohler et al., 2014) (Figure 7Ai). The efficiency of AβO binding to the neuronal surface is strongly reduced in cultured *Prpn−/−* neurons, and AβOs fail to inhibit hippocampal LTP in *Prpn−/−* mice indicating that PrP^c^ is one of the major neuronal receptors for AβOs (Laurén et al., 2009). The AβO-induced LTP impairment is also reduced in rats after intracerebroventricular administration of antibody fragments directed to a putative Aβ-binding site on PrP^c^ (Barry et al., 2011). Furthermore, PrP^c^ shedding due to cleavage by ADAM10 decreases AβO binding to iPSC-derived neurons, resulting in reduced toxicity (Jarosz-Griffiths et al., 2019), while soluble PrP^c^ inhibits AβO-induced LTP inhibition (Calella et al., 2010; Scott-McKean et al., 2016).

Binding of AβO to PrP^c^ leads to an increase in intracellular calcium levels, causes synaptotoxicity, and induces activation of Fyn kinase leading to tau hyperphosphorylation (Larson et al., 2012; Peters et al., 2015). PrP^c^ transmits signals across the plasma membrane by binding to LRP1 (Rushworth et al., 2013) and mGluR5 (Um et al., 2013; Hu et al., 2014). NCAMs, which bind both PrP^c^ and Fyn, may also be involved (Bodrikov et al., 2005; Santuccione et al., 2005; Wang et al., 2013). Binding of AβO to PrP^c^ has been shown to hinder PrP^c^-dependent BACE1 inhibition, likely promoting further Aβ production and accumulation (Rushworth et al., 2013). Binding of AβO to PrP^c^ at the cell surface leads to an increase in PrP^c^ levels here by limiting PrP^c^ endocytosis (Caetano et al., 2011), thereby further promoting the binding of AβO to the cell surface and enhancing AβO toxicity in a positive feedback loop.

An orally administered PrP^c^ agonist that blocks binding of AβOs to PrP^c^ rescues memory impairment and synaptic loss in transgenic APP/PS1 mice (Gunther et al., 2019), which overexpress human APP with a Swedish mutation and mutant PS1 and exhibit an age-dependent accumulation of AβOs and AβO-induced pathology. The effects of the PrP^c^ agonist are seen in 12-month-old mice, when Aβ accumulation has already occurred, and memory impairment and synaptic loss are evident (Gunther et al., 2019). These data suggest that therapeutic interventions targeting the PrP^c^-AβO interaction may prove effective at restoring brain health in individuals already diagnosed with AD.

### CAMs of the leucine-rich repeat superfamily

CAMs of the leucine-rich repeat superfamily are characterized by the presence of leucine rich-repeats (LRRs) in their extracellular domains. Leucine-rich repeat transmembrane proteins (LRRTMs) are a family within the LRR superfamily consisting of four members (LRRTM-1 to -4). They are single-pass transmembrane proteins with short cytoplasmic tails and extracellular domains comprising ten LRRs (Figure 7Bi). LRRTMs mediate synaptic adhesion by binding to neurexins (Ko, 2012). A family of fibronectin leucine-rich repeat transmembrane (FLRT) proteins also belongs to the LRR superfamily. Three members of this family (FLRT-1 to -3) are single-pass transmembrane proteins with ten extracellular LRR domains and a juxtamembrane Fn3 domain (Figure 7Bii). FLRTs mediate synaptic adhesion by binding to latrophilins (O'Sullivan et al., 2012). LRRTMs and FLRTs play an important role in synapse formation and regulation (Ko, 2012; Schroeder and de Wit, 2018).

#### LRR superfamily CAMs in AD and their role in the amyloidogenic processing of APP

LRRTM3 was identified as a candidate gene for AD from an siRNA screening of over 15,000 genes (Majercak et al., 2006). SNPs within the promoter and intronic regions of the gene coding for LRRTM3 are associated with AD (Reitz et al., 2012). LRRTM3 interacts with APP in HEK293 cells and colocalizes with APP in cultured neurons (Lincoln et al., 2013). siRNA-mediated knockdown of LRRTM3 leads to a reduction in sAPPβ and β-CTF levels and Aβ secretion in SH-SY5Y cells (Majercak et al., 2006; Lincoln et al., 2013), suggesting that LRRTM3 modulates β-cleavage of APP (Figure 7Bi). APP also interacts with FLRT1 and FLRT3 in HEK293T cells (Yu et al., 2016) (Figure 7Bii). The functional role of this interaction remains unclear.

## Conclusions and outlook

The amyloid hypothesis places APP and Aβ at the center of AD etiology, however, our understanding of the normal functions of APP, regulation of its processing, and the mechanisms of Aβ-induced toxicity are still incomplete. These knowledge gaps have been showcased by the failures of therapeutics targeting APP processing and Aβ, which have been unable to demonstrate reasonable efficacy (Kumar et al., 2018; Hampel et al., 2021; Karran and De Strooper, 2022). While the physiological functions of APP are incompletely understood, its general role in regulating cell adhesion is suggested by multiple reports showing that APP not only interacts with different CAMs, but also regulates multiple functions of these proteins, including cell adhesion and neuronal growth regulation. CAMs also emerged as important regulators of the processing of APP with some CAMs promoting amyloidogenic pathway and others enhancing the non-amyloidogenic pathway. In addition to its role in AD pathogenesis, Aβ plays physiological roles in learning and memory (Puzzo et al., 2011; Kent et al., 2020). It is therefore possible that the CAM-regulated switches in the modes of APP processing are functionally important, however, the roles these switches play in the healthy brain remains poorly understood and should be analyzed in the future. Substantial evidence indicates that CAMs function as the cell surface receptors for Aβ and its oligomers. While a possible physiological role of these interactions remains also unknown, the binding of Aβ to CAMs clearly plays a role in AD by inducing aberrant signaling pathways and loss of CAM-mediated adhesion. Interestingly, some CAMs inhibit the formation of large Aβ oligomers, suggesting these proteins are involved in the regulation of Aβ turnover in the brain.

Together, the research outlined here highlights the importance of the interplay between CAMs and APP during normal physiology, a factor which should be considered when developing therapeutics that target APP as they may impact these functions. On the other hand, modulation of the interactions between APP and CAMs may represent an attractive therapeutic approach to reduce Aβ generation while limiting side effects due to overzealous obstruction of APP and its proteases. Finally, inhibition of the binding of Aβ to CAMs may be used to prevent the binding of Aβ to the neuronal surface and reduce the Aβ-induced toxic effects. There are still major gaps in our understanding of the role that CAMs play in APP and Aβ-dependent functions. Future research should focus on the mechanisms underpinning the interactions of CAMs with APP and Aβ in the brain with the hope of aiding future endeavors to develop safe and effective therapies for AD.
